# Assessing the Need for Repeat EEG in Pediatric Patients with Idiopathic Generalized Epilepsy After Anti-Seizure Medication Withdrawal Following Seizure Freedom

**DOI:** 10.1177/08830738241292836

**Published:** 2024-12-10

**Authors:** Sita Paudel, Madison Heebner, Gayatra Mainali, Jaclyn S. Tencer, Rhea Kanwar, Katherine Martel, Ashutosh Kumar, Sunil C. Naik, Sandeep Pradhan, Prakash Kandel, Douglas Leslie

**Affiliations:** 1 Department of Pediatrics and Neurology, 509393 Penn State Health Children's Hospital; 2 College of Medicine, Hershey, PA, USA; 3 Penn State Health, 12311 Penn State Health Children's Hospital

**Keywords:** antiepileptic drugs, antiseizure drugs, EEG, electroencephalography, epilepsy

## Abstract

**Background:**

Most patients with idiopathic generalized epilepsy have good seizure control on antiseizure medications. Although idiopathic generalized epilepsy subtypes such as juvenile absence epilepsy and juvenile myoclonic epilepsy have a high risk of relapse, childhood absence epilepsy may have seizure remission. After 2 years of seizure freedom in childhood absence epilepsy, typically antiseizure medications are discontinued, but follow-up protocols are unclear. This study aims to evaluate how often patients with idiopathic generalized epilepsy undergo electroencephalography (EEG) after antiseizure medication withdrawal, how often antiseizure medications are restarted based on EEG findings, and if this varies between physicians and advanced practice providers at our institution.

**Methods:**

This was a retrospective chart review. Data were collected using electronic medical records of pediatric patients (<18 years) with idiopathic generalized epilepsy who were successfully weaned off antiseizure medications at Penn State Children's Hospital from 2010 to 2020.

**Results:**

We reviewed 1409 charts and found 52 patients meeting criteria. Seventeen of 52 patients (32%) had a repeat EEG within 6 months of antiseizure medication withdrawal following seizure freedom. Of those 17 patients, 3 (17.6%) had generalized epileptiform discharges on EEG. Of these 3 patients, 2 (66%) were restarted on antiseizure medications based on the abnormal EEG. None had seizure relapse.

**Conclusion:**

Obtaining a repeat EEG in patients after antiseizure medication withdrawal following seizure freedom is common. Patients with an abnormal EEG are often restarted on antiseizure medications, irrespective of clinical seizure relapse. Considering the high health care costs of EEGs and antiseizure medication side effects, we propose that if patients with idiopathic generalized epilepsy do well clinically following antiseizure medication withdrawal, EEGs may not be necessary.

Epilepsy is defined as the propensity to experience recurrent seizures.^1^ Idiopathic generalized epilepsy is an epilepsy subtype composed of multiple epileptic syndromes including childhood absence epilepsy, juvenile absence epilepsy, juvenile myoclonic epilepsy, and epilepsy with generalized tonic-clonic seizures alone.^2^ Each of these seizure syndromes have varying prognoses and rates of resolution, in addition to marked individual patient variation. Although some subtypes such as juvenile absence epilepsy and juvenile myoclonic epilepsy carry a high risk of relapse, patients with childhood absence epilepsy frequently experience seizure remission and become seizure free in early adolescence.^3–6^ These patients are often weaned off daily antiseizure medications after two years of seizure freedom.^6^

Previous studies have suggested that the persistence of epileptiform discharges on electroencephalogram (EEG) do indicate a higher risk of seizure recurrence, and therefore may caution the provider against weaning medications.^7–15^ However, continuing medications in patients with clinical seizure freedom is not always the optimal treatment. In addition, once antiseizure medications are removed, the risk of seizure recurrence varies from patient to patient.^16^ There is no clear consensus regarding whether an EEG with persistent epileptiform discharges in the absence of clinical seizures signals the need for medication reinitiation.^17,18^ There is also a general lack of standardized guidance for following patients after medication withdrawal, and no agreement on if a repeat EEG is even needed following antiseizure medication wean in the absence of clinical seizures. As a result, there is significant provider variation in management of these patients.

An abnormal EEG does not always translate to high risk for seizures. A study by Giel et al^19^ reported abnormal EEG activity in 22% of patients with migraines and in 24% of patients suffering from psychogenic or tension headaches; additionally, some of the healthy male patients within their control group demonstrated unspecified abnormal EEG findings, and a relatively consistent percentage of borderline EEGs was found within all 3 groups.

The lack of specificity of an abnormal EEG may lead to reintroduction of antiseizure medications for patients who are otherwise clinically well and without seizure relapse. Although antiseizure medications can be helpful in reducing or eliminating seizures and preventing seizure recurrence, they can also be associated with several adverse effects and poor long-term outcomes such as neurodevelopmental issues.^20^ Furthermore, there are additional clinical considerations for patients on antiseizure medications who are able to or desire to become pregnant, as some of the antiseizure medications such as valproic acid have the potential to be teratogenic.^21^

Identifying the need for repeat EEGs and elucidating the association between an abnormal EEG post-antiseizure medication withdrawal and the rate of seizure relapse in patients with idiopathic generalized epilepsy, especially childhood absence epilepsy, could assist in the development of clearer guidance for management of this large population of patients. The objectives of this study are primarily to assess the need for repeat EEG following seizure medication withdrawal in children with generalized epilepsy, and also to analyze potential differences in practice pattern between advanced practice providers and physicians within our institution.

## Methods

This was a retrospective chart-review study completed after Institutional Review Board (IRB) approval. Data collection was completed by filtering charts using *International Classification of Diseases, Tenth Revision*, codes and compiling with TriNetX, a research population cohort search tool. The TriNetX database is a global health-collaborative clinical research network that collects and accesses comprehensive electronic medical records, including diagnoses, procedures, medications, laboratory values, and genomic information from large health care organizations. The data collected and displayed from this platform undergoes a deidentification process to protect the privacy and security of the data per the standard defined in section 164.514(a) of the Health Insurance Portability and Accountability Act privacy rule (Publication Guidelines, TriNetX). This search yielded 1409 individual electronic medical records of children (<18 years) with a history of idiopathic generalized epilepsy at Penn State Children's Hospital. Medical records from 2010 to 2020 were pulled for this study. To confirm the diagnosis of idiopathic generalized epilepsy, diagnostic data including clinical history and EEG reports were reviewed. Data from patients with idiopathic generalized epilepsy were collected to determine whether these patients were currently on antiseizure medications, if they had been previously weaned off antiseizure medications, if they had an EEG performed before or after antiseizure medication weaning, if there were abnormal EEG findings, and if the patient experienced seizure relapse.

Inclusion criteria were defined as children <18 years of age with a diagnosis of idiopathic generalized epilepsy who had EEG findings of generalized spike and wave complexes with otherwise normal background and who were weaned off antiseizure medications following seizure freedom. Exclusion criteria were defined as children with other types of epilepsy, provoked seizures such as a febrile illness or trauma, those who stopped antiseizure medications against medical advice, those with incomplete chart data, or those who were lost to follow-up. Patients who received an EEG after antiseizure medication weaning for reasons such as follow-up after a seizure or suspected seizure were excluded, as these patients did not achieve technical seizure freedom.

Demographic and clinical characteristics were summarized using means and standard deviations (SDs) for continuous variables and counts and percentages for categorical variables of the study group. Because the data were not normally distributed after evaluating continuous data for homogeneity of variance and normal distribution, the quantitative variables were summarized using nonparametric Wilcoxon rank-sum tests and Fisher exact tests to determine the difference in demographic characteristics, EEG findings, and clinical findings for various outcomes including those who had repeat EEGs versus those who did not have repeat EEGs. A probability level of *P* = .05 (2-tailed) was used to determine the statistical significance of group differences. Histograms were used to compare the differences between groups visually. Because this is a descriptive report, statistics and resulting probability values are to be taken as descriptive indicators, not inferential ones.

The statistical analyses were performed by using SAS, version 9.00 (SAS Institute Inc, Cary, NC).

## Results

**Figure 1.**
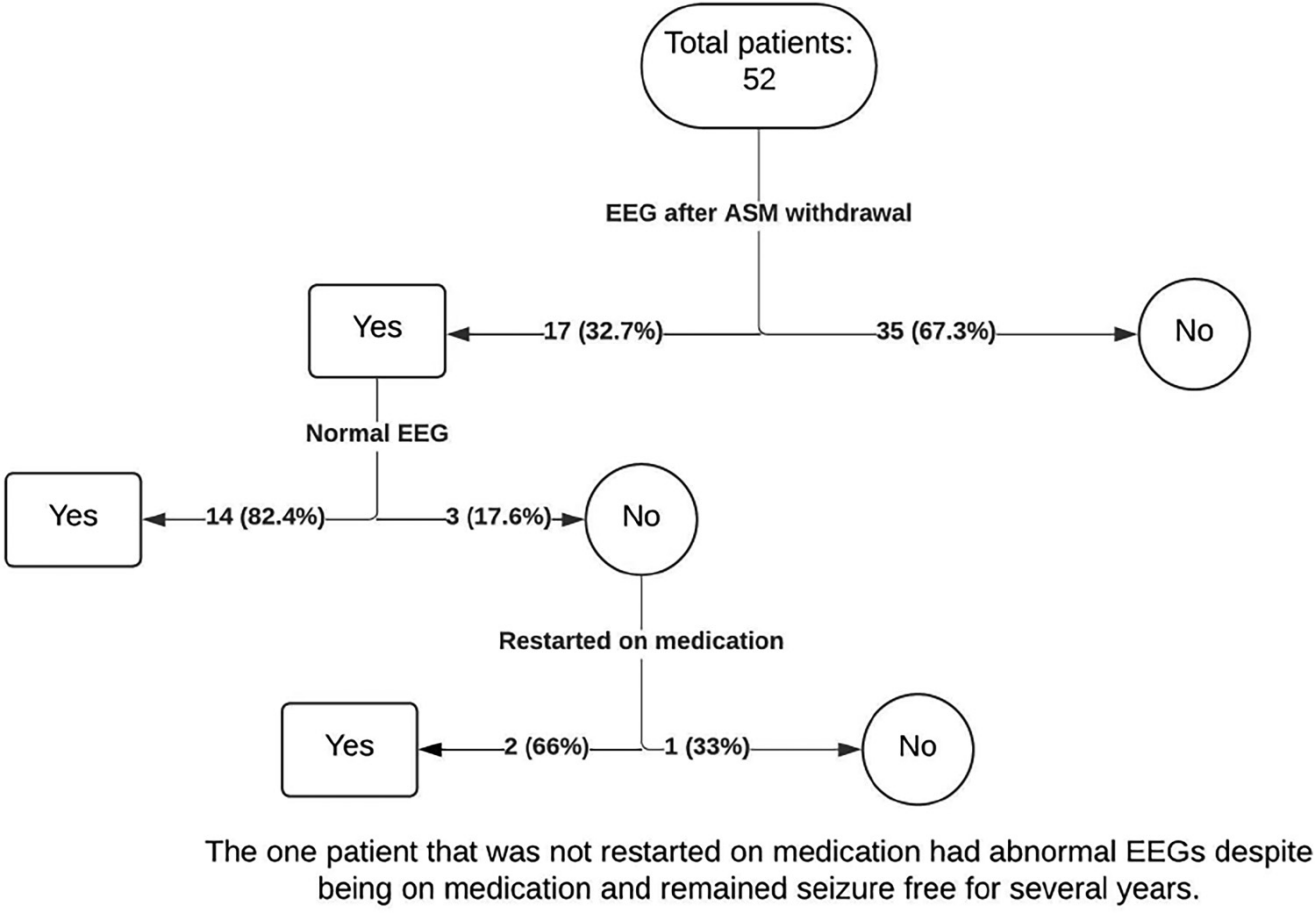
Patient demographics flowchart. ASM, antiseizure medication; EEG, electroencephalography.

We reviewed 1409 charts and found 52 patients who met our inclusion criteria (Table 1, Figure 1). The median age of epilepsy onset was 6 years, which was the same as the median age when antiseizure medications were begun. Seizure medications were withdrawn at a median age of 9 years. All patients had generalized epilepsy with generalized spike and wave discharges on their EEG at the time of diagnosis. Thirty-one patients (59.6%) were diagnosed with unspecified idiopathic generalized epilepsy (idiopathic generalized epilepsy non–childhood absence epilepsy), and 21 (40.4%) were diagnosed with childhood absence epilepsy, specifically.

**Table 1.** Patient Demographics (N = 52).

Characteristic	Value
Age of epilepsy onset	
Mean (SD)	6.4 (3.03)
Median	6.0
Range	1.0, 13.0
Age when ASMs were started	
Mean (SD)	6.5 (2.98)
Median	6.0
Range	1.0, 13.0
Age when ASM was withdrawn	
Mean (SD)	9.5 (2.85)
Median	9.0
Range	4.0, 16.0
Gender, n (%)	
Female	19 (36.5)
Male	33 (63.5)
EEG findings prior to drug withdrawal, n (%)	
Normal	49 (94.2)
Abnormal	1 (1.9)
Not Obtained	2 (3.8)
Types of ASMs, n (%)	
Ethosuximide	23 (44.2)
Levetiracetam	11 (21.2)
Valproic acid	10 (19.2)
Lamotrigine	3 (5.8)
Topiramate	1 (1.9)
Polytherapy	4 (7.7)
Duration of treatment, n (%)	
<3 y	30 (57.7)
≥3 y	22 (42.3)
EEG obtained after ASM withdrawal, n (%)	
No	35 (67.3)
Yes	17 (32.7)
Patient restarted on ASM following EEG, n (%)	
No	15 (88.2)
Yes	2 (11.8)
Repeat electroencephalogram, n (%)	
Normal	14 (82.4)
Abnormal	3 (17.6)
APP vs MD/DO, n (%)	
APP	34 (65.4)
MD/DO	18 (34.6)
IGE syndrome, n (%)	
CAE	21 (40.4)
IGE non-CAE	31 (59.6)

Abbreviations: APP, advanced practice provider; ASM, antiseizure medication; CAE, childhood absence epilepsy; EEG, electroencephalography; IGE, idiopathic generalized epilepsy; MD/DO, physician.

Prior to antiseizure medication withdrawal, 50 patients (96.2%) underwent EEG, of which 1 (2.0%) was abnormal. This patient consistently had abnormal EEGs despite being on antiseizure medications and remaining seizure-free for several years, and the decision was made to withdraw antiseizure medications despite the abnormal EEG. Two patients (3.9%) did not receive an EEG prior to antiseizure medication withdrawal for unknown reasons. After antiseizure medication withdrawal following seizure freedom, 17 of 52 patients (32.7%) had a repeat EEG obtained within 6 months to monitor for epileptiform activity or other unspecified abnormalities (Table 2). Of those 17 patients, 3 (17.6%) had abnormal EEG findings such as generalized epileptiform discharges (Table 2). Of these 3 patients, 2 (66%) were restarted on antiseizure medications based on unspecified abnormal EEG findings. None had clinical seizure relapse as of the date of last chart review, which took place in December 2023 (Table 2), more than 1 year since discontinuing the antiseizure medications in 2022.

**Table 2.** Patient Demographics by EEG findings.

	Repeat electroencephalogram			
	Normal (n = 14)	Abnormal (n = 3)	Total (n = 17)	*P* value
Age of epilepsy onset				.3372^a^
Mean (SD)	6.9 (3.22)	5.7 (2.31)	6.7 (3.06)	
Median	8.0	7.0	7.0	
Range	1.0, 12.0	3.0, 7.0	1.0, 12.0	
Age when ASMs were started				.2200^a^
Mean (SD)	7.2 (3.12)	5.7 (2.31)	6.9 (2.99)	
Median	8.0	7.0	8.0	
Range	1.0, 12.0	3.0, 7.0	1.0, 12.0	
Age when ASM was withdrawn				.4824^a^
Mean (SD)	10.1 (3.38)	9.0 (1.00)	9.9 (3.10)	
Median	10.0	9.0	10.0	
Range	4.0, 16.0	8.0, 10.0	4.0, 16.0	
Gender, n (%)				>.9999^b^
Female	7 (50.0)	2 (66.7)	9 (52.9)	
Male	7 (50.0)	1 (33.3)	8 (47.1)	
EEG findings at the time of diagnosis, n (%)				
Generalized spike and wave	14 (82.4)	3 (17.6)	17 (100.0)	
EEG findings prior to drug withdrawal, n (%)				.1765^b^
Normal	14 (87.5)	2 (12.5)	16 (94.1)	
Abnormal	0 (0.0)	1 (100.0)	1 (5.9)	
Type of epilepsy, n (%)				
Generalized	14 (82.4)	3 (17.6)	17 (100.0)	
Type of ASMs, n (%)				>.9999^b^
Ethosuximide	6 (75.0)	2 (25.0)	8 (47.1)	
Levetiracetam	3 (100.0)	0 (0.0)	3 (17.6)	
Valproic acid	3 (75.0)	1 (25.0)	4 (23.5)	
Lamotrigine	1 (100.0)	0 (0.0)	1 (5.9)	
Topiramate	1 (100.0)	0 (0.0)	1 (5.9)	
Duration of treatment, n (%)				>.9999^b^
<3 y	7 (87.5)	1 (12.5)	8 (47.1)	
≥3 y	7 (77.8)	2 (22.2)	9 (52.9)	
EEG obtained after ASM withdrawal, Y/N, n (%)				
Yes	14 (82.4)	3 (17.6)	17 (100.0)	
Patient restarted on ASM, n (%)				.0221^b^
No	14 (93.3)	1 (6.7)	15 (88.2)	
Yes	0 (0.0)	2 (100.0)	2 (11.8)	
APP vs MD/DO, n (%)				>.9999^b^
APP	7 (77.8)	2 (22.2)	9 (52.9)	
MD	7 (87.5)	1 (12.5)	8 (47.1)	
IGE syndrome, n (%)				.5368^b^
CAE	5 (71.4)	2 (28.6)	7 (41.2)	
IGE non-CAE	9 (90.0)	1 (10.0)	10 (58.8)	

Abbreviations: APP, advanced practice provider; ASM, antiseizure medication; CAE, childhood absence epilepsy; EEG, electroencephalography; IGE, idiopathic generalized epilepsy; MD/DO, physician.

^a^ Wilcoxon rank-sum *P* value.

^b^ Fisher exact *P* value.

Within the patient population, some patients primarily saw an advanced practice provider and some patients saw a physician for their follow-up appointments. Thirty-four patients (65.3%) primarily saw an advanced practice provider and 18 (34.6%) primarily saw a physician. Of the 34 patients who saw an advanced practice provider, a repeat EEG was obtained in 9 (26.5%), and of these 9 patients, 2 (22.2%) had an abnormal EEG. Of the patients who saw a physician, a repeat EEG was obtained in 8 (44.4%) and 1 (12.5%) of these was abnormal. Of the patients who did have an abnormal EEG, 1 advanced practice provider patient (11.1%) and 1 physician patient (12.5%) were restarted on antiseizure medications. The majority of the advanced practice provider–treated patient population had a treatment duration of <3 years (22 patients, 64.7%), whereas the majority of the physician-treated patient population had a treatment duration of ≥3 years (10 patients, 55.6%).

**Figure 2.**
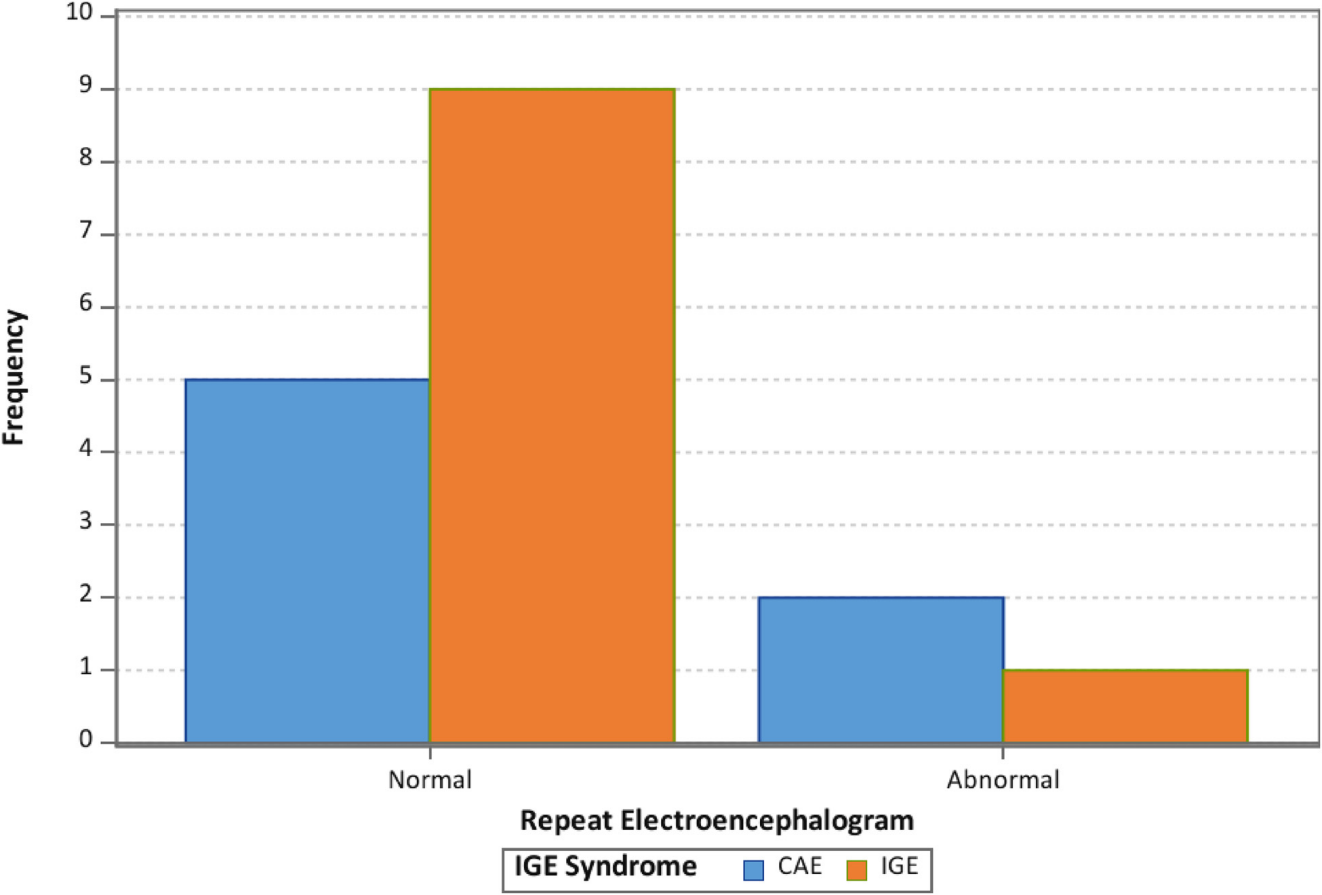
Histograms for rate of repeat electroencephalogram (EEG) by idiopathic generalized epilepsy syndrome. CAE, childhood absence epilepsy; IGE, idiopathic generalized epilepsy.

**Figure 3.**
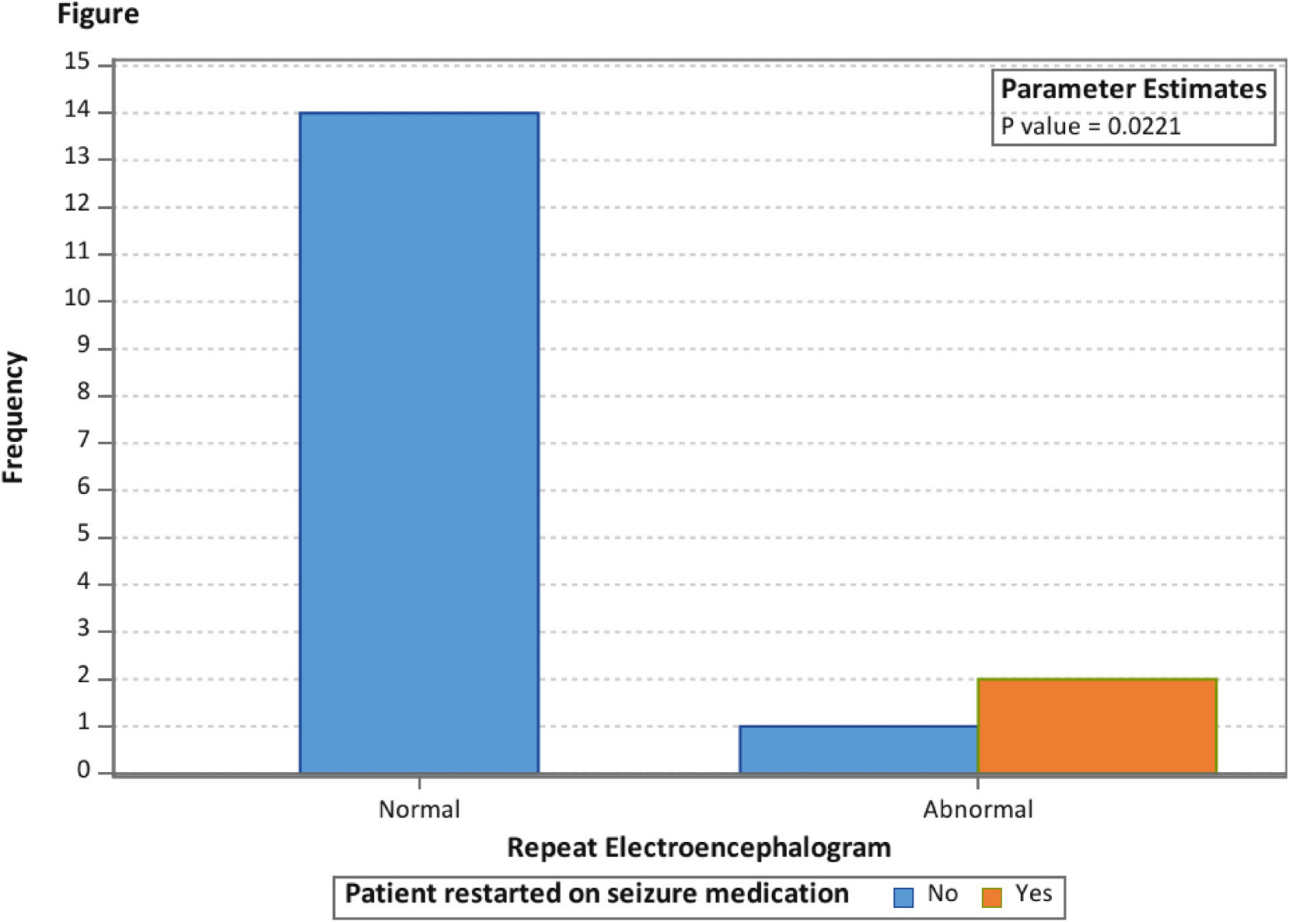
Rate of repeat electroencephalogram (EEG) by patients restarted on antiseizure medication.

Overall, we did not find a statistical significance in the rate of EEG abnormalities on follow-up EEGs when comparing patients with childhood absence epilepsy and idiopathic generalized epilepsy non–childhood absence epilepsy (Table 2, Figure 2). There was a statistically significant difference (*P* = .0221) in the frequency of restarting antiseizure medications in patients with a normal follow-up EEG when compared to patients with an abnormal follow-up EEG, where zero of 14 patients with a normal EEG were restarted on antiseizure medications and 2 of 3 patients (66%) with an abnormal EEG were restarted on antiseizure medications (Table 2, Figure 3).

**Figure 4.**
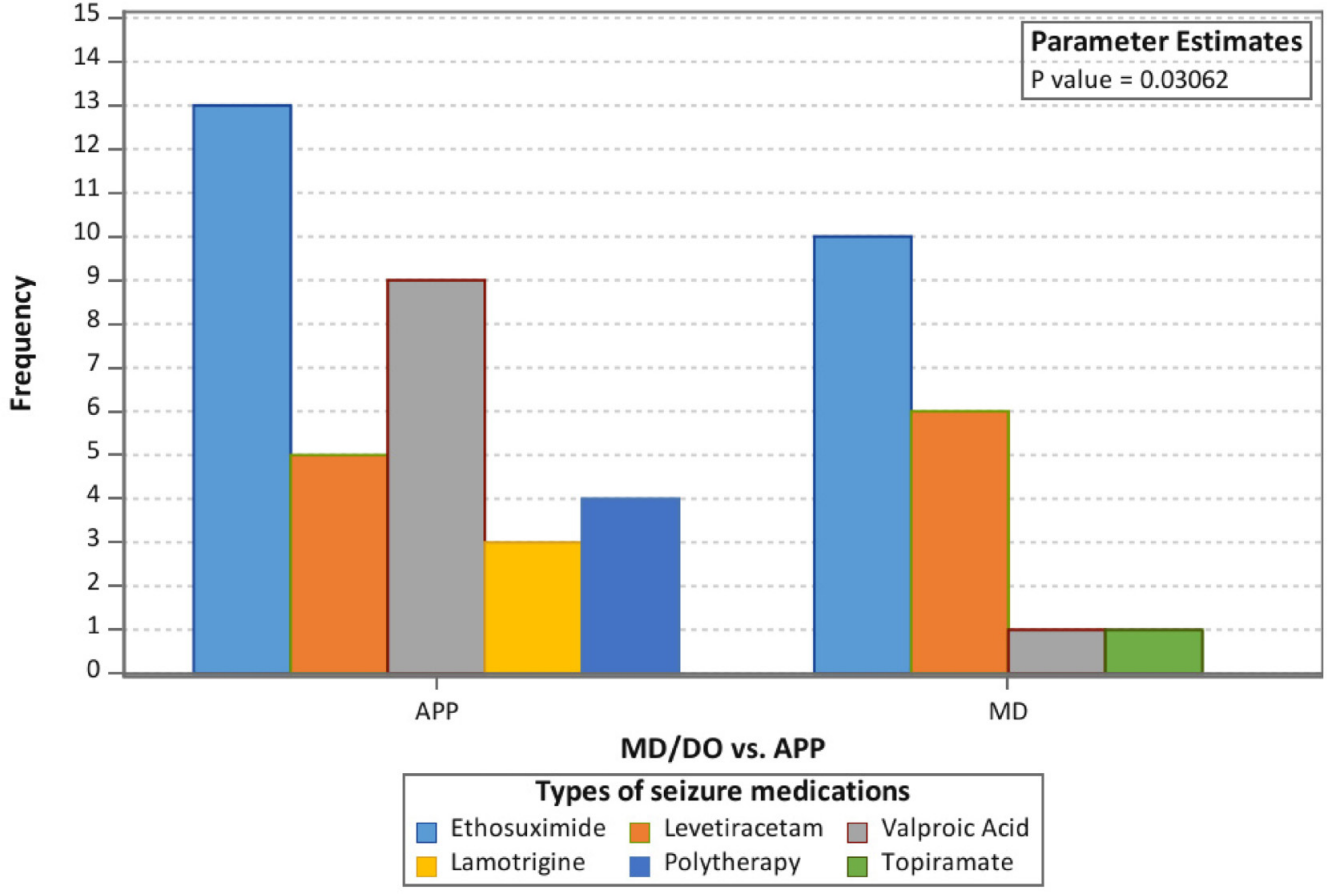
Frequency of antiseizure medications started by physician (MD/DO) vs advanced practice provider (APP) prior to Withdrawal.

Additionally, there was also a statistically significant difference (*P* = .03062) in the antiseizure medication type used when comparing advanced practice provider with physician patient populations (Figure 4), although there was no significant difference in the rate of prescription of any 1 medication specifically. Prior to withdrawal, patients were maintained on a variety of medications, including monotherapy with ethosuximide, levetiracetam, valproic acid, lamotrigine, topiramate, or a combination of antiseizure medications (polytherapy). Twenty-three patients (44.2%) were maintained on ethosuximide. Of these 23 patients, 13 (56.5%) primarily saw an advanced practice provider, and 10 (43.5%) primarily saw a physician. Eleven patients (21.2%) were maintained on levetiracetam, 5 (45.5%) of which primarily saw an advanced practice provider and 6 (54.5%) of which saw a physician. Ten patients (19.2%) were maintained on valproic acid, 9 (90.0%) of which primarily saw an advanced practice provider and 1 (10.0%) of which primarily saw a physician. The remaining patients were maintained on lamotrigine, topiramate, or a combination of antiseizure medications.

## Conclusion

Obtaining one or more repeat EEGs in pediatric patients with idiopathic generalized epilepsy after withdrawal of seizure medication following seizure freedom is not rare. It is also not rare to identify abnormal findings when an EEG is obtained. Antiseizure medications are often restarted if an EEG shows abnormal findings, even in the absence of seizure relapse. This practice is likely due to results of the previous studies by Yıldırım et al^20^ that showed increased risk of seizure relapse in patients with abnormal EEG. Another study, by Huang et al,^21^ also concluded that abnormal EEG after drug withdrawal is a risk factor for the recurrence of epilepsy in children.

Similarly, within our institution, the trend to restart medication after an abnormal EEG was present in both physicians and advanced practice providers. However, unnecessary EEGs can be associated with high health care costs and overutilization, and antiseizure medications can have notable side effects, such as possibility of poor neurodevelopmental outcomes, adverse effects during treatment, and potential teratogenic effects.^22–24^

Therefore, we suggest that if patients with idiopathic generalized epilepsy, especially childhood absence epilepsy, are doing well clinically following antiseizure medication withdrawal, an EEG may not be required. However, our study is limited because of the small sample size and single center studied, and the limited opportunity for long-term follow-up to monitor for seizure relapse. Therefore, a larger study completed over a longer course and with randomized controlled trials would be necessary to further evaluate the utility of EEGs in this patient population, to ensure that patients with abnormal EEGs in follow-up would not experience clinical seizure relapse in the future.
